# Are People with Aphasia Included in Stroke Trials? A Systematic Review and Narrative Synthesis

**DOI:** 10.1177/02692155231172009

**Published:** 2023-05-15

**Authors:** Eileen Vaughan, Molly X Manning

**Affiliations:** 1 Department of Speech and Language Therapy, University Hospital Limerick, Limerick, Ireland; 2 School of Allied Health, University of Limerick, Co. Limerick, Ireland; 3 Public and Patient Involvement Research Unit, Health Research Institute, University of Limerick, Co. Limerick, Ireland

**Keywords:** Stroke, aphasia, systematic reviews, randomised controlled trials, health services research

## Abstract

**Objective:**

To examine the proportion of people with aphasia (PwA) included and retained in randomised controlled trials (RCTs) of stroke interventions published in the previous 6 years, as well as aphasia-relevant eligibility criteria and inclusion/retention strategies.

**Data sources:**

Comprehensive searching of Embase, PubMed and Medline (Ovid) for the period January 2016 – November 2022.

**Review methods:**

RCTs examining stroke interventions targeting cognition, psychological wellbeing/health-related quality of life (HRQL), multidisciplinary rehabilitation, and self-management were included. Methodological quality was assessed using the Critical Appraisal Skills Programme (CASP) Randomised Controlled Trial checklist. Descriptive statistics were applied to extracted data, and results were reported narratively.

**Results:**

Fifty-seven RCTs were included. These examined self-management (32%), physical (26%) psychological wellbeing/HRQL (18%), cognitive (14%), and multidisciplinary (11%) interventions. Of 7313 participants, 107 (1.5%) had aphasia and were included in three trials. About one-third did not report on aphasia (32%); over one quarter required functional communication (28%); one quarter excluded all aphasia (25%); and 14% excluded severe aphasia. No aphasia-specific inclusion/retention strategies were available.

**Conclusion:**

The findings highlight ongoing under-representation. However, due to shortcomings in aphasia reporting, the findings may underestimate actual inclusion rate. Excluding PwA has implications for the external validity, effectiveness, and implementation of stroke research findings. Triallists may require support in aphasia research strategies and methodological reporting.

## Introduction

In stroke research, people with aphasia are commonly under-represented in, and often excluded from, intervention trials for which language status is highly salient including post-stroke information, depression screening, and self-management.^1–4^ They are better represented in trials of early acute interventions that may have exemption from informed consent directives.^5^ Consequently, a growing body of online, English-language resources is freely available to equip triallists in including and retaining individuals with aphasia, for example, by developing accessible study materials, dissemination reports, and informed consent processes.^6–9^

The exclusion of people with aphasia impacts on the representativeness of stroke trial findings with implications for equity and quality of clinical management.^10^ Equipping stroke triallists to include people with aphasia is important for efficient use of trial resources and to more validly inform clinical treatment and care decisions.^11,12^ It is unclear, however, if these initiatives have yet translated into improved representation and consideration of aphasia in stroke trials. The purpose of this study, therefore, was to conduct a systematic review of recent stroke trials to examine the rates of inclusion and retention of people with aphasia and use of aphasia-friendly inclusion and retention strategies. We focused only on interventions likely to be impacted by aphasia-status, for example, in trial conduct and real-world implementation. Therefore, we examined only trials of interventions targeting psychological function, cognition, multidisciplinary rehabilitation, and self-management, published in the previous six-year period. We asked the following research questions: (1) What proportion of participants included and retained have aphasia? (2) What inclusion and retention strategies specific to aphasia are described? (3) What eligibility criteria relevant to aphasia and/or communication are given?

## Methods

We registered the protocol for this systematic review with the International Prospective Register of Systematic Reviews (PROSPERO) CRD42021264295^13^ and followed the Preferred Reporting Items for Systematic Reviews and Meta-Analyses 2020 checklist (Supplementary file 1).^14^ We developed a comprehensive search strategy informed by scoping searches to identify key articles and search terms. The search strategy targeted articles published in English in peer-reviewed journals since January 2016 that reported data from quantitative randomized controlled trial studies (RCTs) using comparative conditions. Studies were included only if the participants were all adults with stroke aged 18 years and above. Some exclusions were applied based on the intervention focus because we reasoned that certain interventions would be unlikely to be impacted by aphasia and communication status. For example, communication ability might be a prerequisite for partaking in interventions incorporating a dialogue-based component. We therefore excluded medical, surgical, and pharmacological interventions, including Botox, needling, electromyography, brain stimulation, and secondary prevention. Non-dialogue-based interventions without a primary focus on cognition, health-related quality of life, psychological wellbeing, and/or self-management were also excluded. These include physical interventions like core stability, weight shift training, limb function, gait, or dysphagia. Finally, because we wanted to examine the inclusion of people with aphasia in stroke trials, we excluded RCTs of aphasia and/or communication-specific interventions. The full eligibility criteria are in Appendix 1.

The searches were conducted in June 2021 and in November 2022.

In June 2021, the first author searched three electronic databases, EMBASE, PubMed, and MEDLINE (Ovid), downloaded results to EndNoteX9 and removed duplicates. The processes of citation screening, data extraction, and syntheses were led by the first author in close consultation with the final author. Both authors independently screened title and abstract for the first 100 citations against eligibility criteria in Appendix 1. Discrepancies were resolved through discussion. The first author completed the remaining title/abstract screening independently and led full-text screening. The second author subsequently repeated the search process to capture articles published from June 2021 – November 2022 in close consultation with the first author. The full search strategy is in Appendix 2.

The following data were extracted for analysis using a pre-prepared data extraction template (Supplementary file 2): intervention; number of participants included (with and without aphasia); proportion retained; and eligibility criteria concerning aphasia or communication status. Descriptive statistics were applied in Excel. Interventions were grouped into similar categories. Any documented inclusion and retention strategies, and author reflections on communication-related eligibility, inclusion and/or retention, were summarised narratively. Critical appraisal was conducted using the CASP (Critical Appraisal Skills Programme) Randomised Controlled Trial Standard Checklist.^15^ This instrument contains eleven questions that are rated on a three-point scale (yes, no, can’t tell) and appraise the reporting of the study design, methodology, results, and usefulness or potential for application. Both authors independently appraised ten articles to ensure inter-rater reliability. At this point, the remaining articles were divided for single appraisal.

## Results

**Figure 1.**
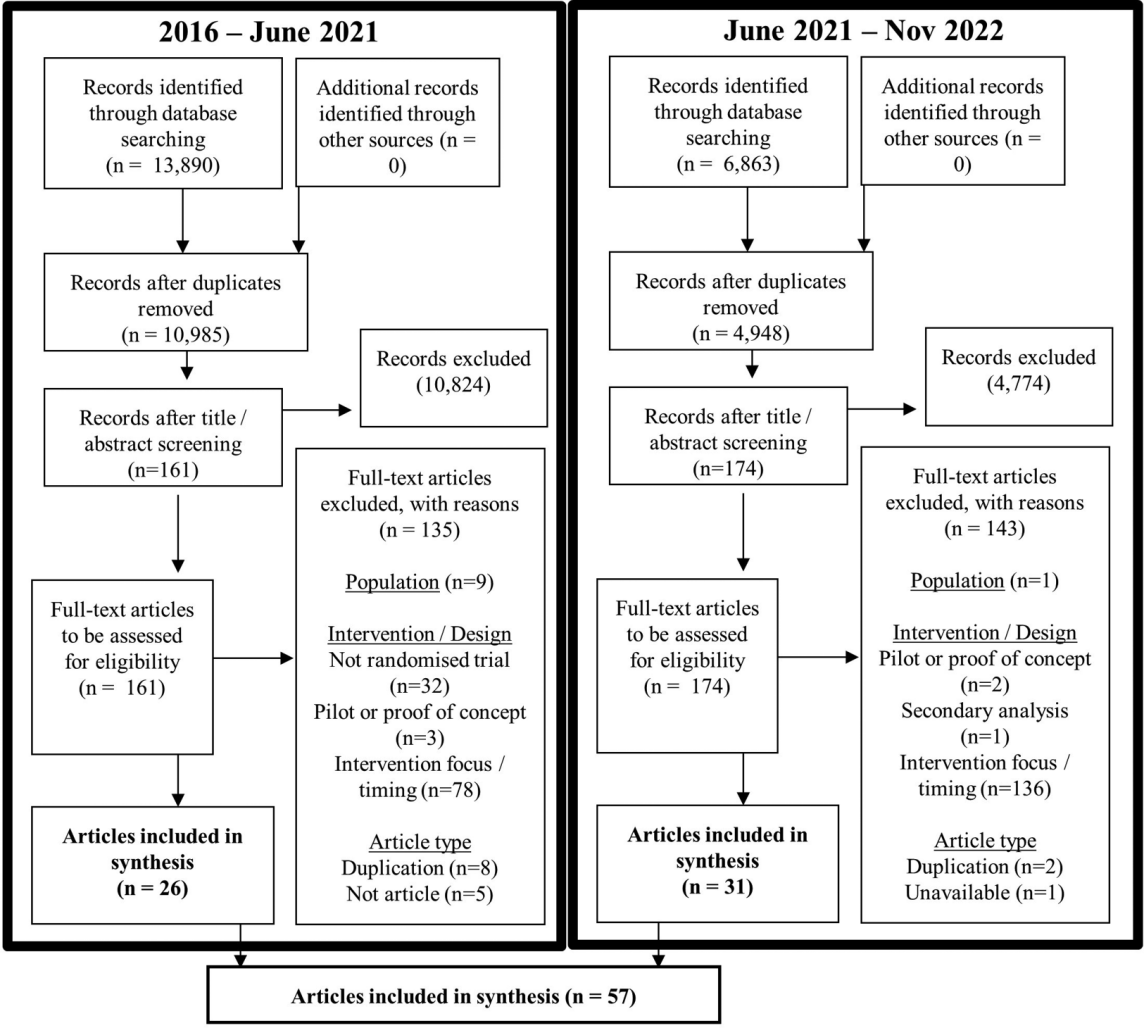
Selection of sources of evidence.

The results of initial electronic database searching yielded 13,890 citations. Following de-duplication, 10,985 citations were screened. Eligibility assessment was conducted for 161 full-text articles. Of these, 135 were excluded: 9 were not specific to stroke; 32 were not randomised controlled studies; 3 were pilot studies or proof of concept; 78 examined effectiveness of excluded intervention types; 8 were duplicates; and 5 were not peer-reviewed journal articles. From initial searching, 26 articles were included. Updated searching in November 2022 yielded 6863 citations and 4949 after de-duplication. Eligibility assessment was conducted for 174 full-text articles. Of these, 143 were excluded: 1 was not specific to stroke; 2 were pilot studies or proof of concept; 1 was a secondary analysis; 136 examined effectiveness of excluded intervention types; 2 were duplicates; and 1 was unavailable through library searching and after contacting the article authors. The updated search generated 31 articles for inclusion. Then, combining articles from the initial and updated searches, a total of 57 articles were included for analysis (PRISMA flow chart,^14^ Figure 1).

A full summary of included studies is in Supplementary File 2. Key aggregated data outlined in the sections below are presented in Table 1.

**Table 1.** Aggregated data obtained from included studies

**Intervention categories *n*, (%)**	
Physical	15 (26)
Cognitive	8 (14)
Self-management	18 (32)
Psychological wellbeing and HRQL	10 (18)
Multidisciplinary rehabilitation	6 (11)
**Participant inclusion and retention**	
Total included in 57 RCTs	7313
Mean included (SD)	128 (35)
RCTs reporting retention number (%)	54 (95)
Total retained in 54 RCTs	6077
RCTs reporting reasons for attrition (%)	39 (68)
Studies reporting inclusion and/or retention strategies (%)	9 (16)
**Aphasia status, *n* (%)**	
Total participants with aphasia	107 (1.5)
Number of trials that included participants with aphasia	3 (5)
**Eligibility criteria relevant to aphasia and communication, n (%)**	
Functional communication description	16 (28)
Excludes severe aphasia	8 (14)
Excludes moderate–severe aphasia	1 (2)
Excludes all aphasia and/or communication problems	14 (24.5)
Aphasia and/or communication criteria are not explicit or are unclear	18 (31.5)

### Intervention Categories

We grouped the interventions into five categories: (1) *Physical* interventions, including a cognitive, dual tasking and/or wellbeing component (n = 15, 26%); (2) *Cognitive* interventions, including attention, memory, reasoning, executive functioning (n = 8, 14%); (3) *Self-management* interventions, including self-efficacy, adherence, health behaviour, and sexual health (n = 18, 32%); (4) *Psychological wellbeing and health-related quality of life (HRQL)* interventions (n = 10, 18%); (5) *Multidisciplinary rehabilitation* interventions, with a focus on any of categories 1–4 (n = 6, 11%). See Table 1.

### Participant Inclusion and Retention

The total number of participants across all 57 trials was 7313; and mean was 128 (SD = 35). The total participants retained across 57 trials was 6077; 83% of participants were originally included. Three studies (5%) did not report retention number. The mean percentage of participants retained in the other 54 studies was 81% (SD = 4). Under one-fifth of trials reported 100% retention (*n* = 10, 18%). Over half of trials (*n* = 39, 68%) documented reasons for participant attrition.

Less than one-fifth of trials (*n* = 9; 16%) reported inclusion and/or retention strategies. Examples of inclusion strategies were involving a proxy, legal guardian, and/or caregiver in the consent process; a physician evaluation prior to participation; additional access to Speech and Language Therapy and/or cognitive rehabilitation alongside the trialed intervention, as relevant; and providing both written and verbal study information. Documented retention strategies included reimbursing travel expenses; providing an honorarium; providing further information if requested to ensure that the control group was not clinically disadvantaged; follow-up stroke nurse advice by phone; support for intervention activities; and specific feedback and general encouragement from the intervention therapist. No aphasia-specific inclusion and/or retention strategies were reported.

### Reporting of the Inclusion of Participants with Aphasia

We grouped eligibility criteria relating to aphasia and/or communication status into five categories: (1) functional communication was required, including, for example, ability to follow and understand verbal instructions (*n* = 16; 28%); (2) severe aphasia excluded, including severe expressive and/or receptive language (*n* = 8; 14%); (3) moderate-severe aphasia excluded (*n* = 1; 2%); (4) all aphasia and/or communication problems excluded (*n* = 14; 24.5%); (5) no explicit aphasia or communication criteria provided with a lack of clarity about whether people with aphasia were included or not (*n* = 18; 31.5%). The latter included trials with eligibility criteria relating to performance on mini-mental state examination (MMSE) and Montreal Cognitive Assessment (MoCA). Only seven (12%) of RCTs had any description of how the presence of aphasia was determined during participant screening. These RCTs used the National Institutes of Health Stroke Scale (NIHSS) Best Language domain; Ullevaal Aphasia Screening Test (UAS); self-report and observation during screening visit; functional independence measure (FIM) Comprehension scale; or a documented clinical diagnosis of aphasia.

Only three trials (5%) explicitly described participants as having aphasia; however, the precise number and proportion of participants with aphasia in each of these RCTs were unclear. The first examined the effectiveness of a dialogue-based intervention to improve psychological wellbeing.^16^ Just under one-quarter of the participants had aphasia (*n* = 86; 24%); however, given a lack of clarity around the nature of communication deficits among participants, it is possible that this figure included some people with dysarthria and no aphasia. The second study examined the effect of exercise on cognitive function.^17^ No aphasia eligibility criteria were reported. At least one-tenth of participants had aphasia (*n* = 5; 10%); however, it is possible that some data from these participants were not included in the final analysis due to having difficulty understanding test instructions. The third trial examined an executive functioning intervention and excluded severe comprehension problems and dyslexia.^18^ Up to 16 people with aphasia and/or dysarthria were included (43%); however, it is not possible to discern the number with aphasia only. Based on the figures reported in these three studies, a maximum of 107 participants with aphasia (1.5%) were included. No aphasia-specific retention data were available across any included study.

Methodological limitations relating to a lack of inclusion of people with aphasia or communication problems were identified by authors in nine studies (16%). These included shortcomings in ability to generalize findings; exclusion of people vulnerable to depression and psychological problems in relevant intervention studies; perceived difficulties enrolling people with severe aphasia in trials; and the potential of aphasia acting as a potential confounder in interpreting assessment and outcome data in trials of cognitive interventions.

### Inclusion of People with Aphasia by Intervention Category

The breakdown of aphasia eligibility criteria by intervention category is in Table 2. In almost one-third of studies, aphasia and/or communication requirement was not clearly reported (*n* = 18; 32%). These include trials examining physical (*n* = 3), cognitive (*n* = 2), self-management (*n* = 8), psychological wellbeing and health-related quality of life (HRQL) (*n* = 3), and multidisciplinary rehabilitation (*n* = 2). Functional communication was an inclusionary criterion in more than one-quarter of trials (*n* = 16, 28%). These included trials that examined physical (*n* = 6), cognitive (*n* = 3), self-management (*n* = 4), psychological wellbeing/HRQL (*n* = 1), and multidisciplinary rehabilitation (*n* = 2). One-quarter of trials excluded people with all aphasia and/or communication problems (*n* = 14; 25%) including physical (*n* = 3), cognitive (*n* = 2), self-management (*n* = 4), psychological wellbeing/HRQL (*n* = 4), and multidisciplinary rehabilitation (*n* = 5). One further psychological wellbeing/HRQL trial excluded those with moderate to severe aphasia.

**Table 2.** Communication eligibility criteria by intervention category

**Functional communication requirement *n*, (%)**	**16 (28)**
Physical	6
Cognitive	3
Self-management	4
Psychological wellbeing and HRQL	1
Multidisciplinary rehabilitation	2
**Severe aphasia excluded *n*, (%)**	**8 (14)**
Physical	3
Cognitive	1
Self-management	2
Psychological wellbeing and HRQL	1
Multidisciplinary rehabilitation	1
**Moderate/severe aphasia excluded *n*, (%)**	**1 (2)**
Psychological wellbeing and HRQL	1
**All aphasia +/or communication problems excluded *n*, (%)**	**14 (24.5)**
Physical	3
Cognitive	2
Self-management	4
Psychological wellbeing and HRQL	4
Multidisciplinary rehabilitation	1
**Aphasia +/or communication not explicit or unclear *n*, (%)**	**18 (31.5)**
Physical	3
Cognitive	2
Self-management	8
Psychological wellbeing and HRQL	3
Multidisciplinary rehabilitation	2

**Figure 2.**
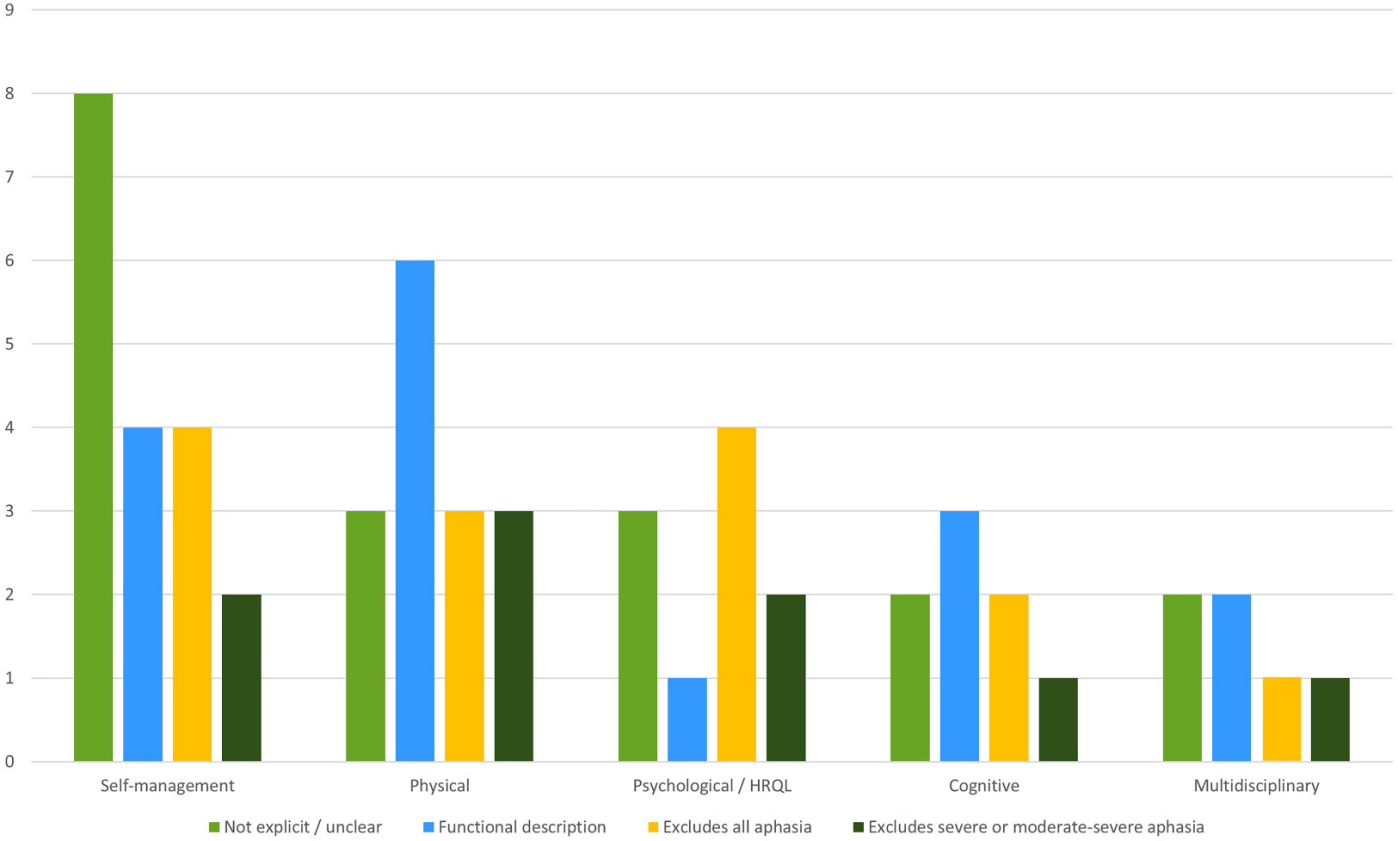
Aphasia eligibility criteria by intervention category.

Of the 18 self-management intervention trials, almost half did not consider aphasia eligibility criteria (*n* = 8; 44%); just under a quarter excluded all aphasia (*n* = 4; 22%) or required certain functional communication ability (*n* = 4; 22%); and two (11%) excluded severe aphasia. Of the 15 physical trials incorporating cognitive or wellbeing outcomes, six (40%) had a functional communication requirement; and the remaining ones either excluded all aphasia (*n* = 3; 20%), excluded severe aphasia (*n* = 3; 20%); or did not refer to aphasia at all (*n* = 3; 20%). Of the 10 psychological/HRQL intervention trials, four excluded all people with aphasia or communication problems (40%); three did not consider communication/aphasia status (30%) one required functional communication (10%), and two excluded severe or moderate-severe aphasia (20%). From eight cognitive interventions, over one third excluded based on functional communication (*n* = 3; 38%); one quarter excluded all aphasia (*n* = 2; 25%); one excluded severe aphasia; and one quarter did not consider aphasia at all (*n* = 2; 25%). Finally, of the six multidisciplinary rehabilitation trials, two did not consider aphasia (33%); two required functional communication (33%); one excluded all aphasia (17%); and one excluded severe aphasia (17%). See Figure 2.

### Critical Appraisal

The basic RCT design was valid in most included studies. However, six (11%) did not address a clearly focused research question; nine (16%) did not clearly report on randomization; and four (7%) did not clearly account for all included participants at conclusion. The methodological quality was less consistent. For most trials, study groups were treated equally apart from the experimental intervention (*n* = 53; 93%). In over half (*n* = 38; 67%), study groups were similar at the outset; however, this was not true of five RCTs (9%) and reporting was unclear in the remaining 14 (25%). Assessors were blinded in over half (*n* = 37; 65%); however, participants and investigators were blind to intervention in only 12 (21%) and 4 (7%), respectively. Results were not consistently reported to a high standard. Although the effects of the intervention were comprehensively reported in 46 (81%); precision of estimate or treatment effect was reported in under half (*n* = 26; 47%); and it was unclear whether the benefits of the intervention outweigh the harms and costs in 45 (79%). Consequently, apart from four studies, most trials were either not applicable to post-stroke aphasia (*n* = 32; 56%) or the potential application was unclear (*n* = 21; 37%). Finally, no experimental intervention was rated as providing greater value to the post-stroke aphasia population compared with existing interventions. See supplementary file 2 for full critical appraisal.

## Discussion

Across 57 stroke trials, three explicitly included people with aphasia; however, precise figures were not clearly reported. Just under one-third did not refer to communication or aphasia in eligibility criteria. Just over one-quarter of RCTs required functional communication (28%); and exactly one-quarter of them excluded all individuals with aphasia and/or communication problems. The remainder excluded people with severe or moderate-severe aphasia (16%). There was no clear pattern of eligibility criteria according to intervention category and aphasia definition and screening processes were unclear. Given that the review sought RCTs of interventions that would likely require adaptation for aphasia, a failure to report on and/or placing recruitment restrictions based on communication status is particularly salient.

The aggregated data support prior reports of a general under-representation and/or reportage of people with aphasia in stroke trials.^1–3,19,20^ This may have important implications for the external validity, effectiveness, and effective implementation of research findings, particularly those which may require adaptation in the context of aphasia. Service providers, policymakers, and researchers must appraise and recognize the inherent limitations of the evidence base accordingly. To enhance recruitment and retention of participants with aphasia and other communication disabilities, stroke researchers may require support and training to engage with and implement accessible research processes and resources,^21^ for example, accessible study materials and consent processes.^6,7,22,23^ It may additionally be useful to examine how best to prioritize or tailor support for trials of stroke interventions that are more likely to require significant adaptation to be clinically effective with people with aphasia. Partnering with people with lived experience throughout the design and conduct of clinical trials through rigorous public and patient involvement or participatory methodologies has been demonstrated to enhance the relevance of the research focus, representativeness and inclusivity of recruitment and retention, and overall quality and generalisability of research outcomes.^24^ This is important for ensuring that trials, which are relatively expensive and resource-intensive, generate relevant, representative participant data that maximize external validity of findings.^25^ Research in this area is essential for designing, conducting, and implementing quality, representative stroke trials that positively impact patient outcomes.

There are several important methodological limitations to be addressed in future research. First, given the timeframe and resources available to the review team, searching was mainly completed by a single author and targeted English language articles only across three databases. Thus, it is possible that relevant studies were omitted. Second, the eligibility criteria of included trials were inspected for reference to aphasia and/or communication status. Although 25% excluded all aphasia, about one-third did not explicitly reference or were unclear about aphasia (32%), and 28% had functional communication requirements, generally around comprehension. Thus, it is possible we have underestimated the inclusion of people with aphasia in stroke trials, and that findings are secondary to the way in which communication ability is reported. Third, due to project resourcing, the scope of the originally registered protocol was reduced^13^ as we were unable to conduct a parallel search and synthesis of aphasia trials to compare inclusion and retention strategies reported, and representative inclusion of participants with severe and moderate aphasia. Thus, under-representation of more vulnerable patient cohorts, and under-reporting of inclusion and retention strategies, may feature in both stroke and post-stroke aphasia trials. Finally, our search strategy excluded trials of interventions that we reasoned would be minimally impacted by communication ability and aphasia including medical, surgical, pharmacological, and physical interventions without cognition or psychological functioning as a key outcome. This has important implications for how our findings should be understood. For example, there is evidence that people with aphasia are well-represented in acute stroke trials,^26^ and so our findings may overstate their exclusion and omit valuable synthesis of inclusionary strategies.

In summary, stroke trials examining a wide range of interventions that are likely to require adaptation in the context of aphasia do not report on aphasia status and often exclude people with aphasia. This may impact on quality, relevance, and acceptability of stroke interventions once implemented into clinical practice. It is essential that stroke service providers, policymakers, and researchers appraise the current evidence base accordingly. Supporting stroke triallists in maximizing inclusion and retention of representative, vulnerable patient cohorts, and in transparently reporting and reflecting on strategies employed is important for improving the future design, conduct, and implementation of stroke trials.

**Clinical messages**

- People with aphasia are under-represented in stroke trials published between 2016 and 2022.
- This has implications for the external validity, effectiveness, and implementation of stroke evidence.
- Stroke triallists may require support to implement inclusive research strategies and to enhance methodological reporting.

## Supplemental Material

sj-pdf-1-cre-10.1177_02692155231172009 - Supplemental material for Are People with Aphasia Included in Stroke Trials? A Systematic Review and Narrative Synthesis Click here for additional data file. Supplemental material, sj-pdf-1-cre-10.1177_02692155231172009 for Are People with Aphasia Included in Stroke Trials? A Systematic Review and Narrative Synthesis by Eileen Vaughan and Molly X Manning in Clinical Rehabilitation

sj-pdf-2-cre-10.1177_02692155231172009 - Supplemental material for Are People with Aphasia Included in Stroke Trials? A Systematic Review and Narrative Synthesis Click here for additional data file. Supplemental material, sj-pdf-2-cre-10.1177_02692155231172009 for Are People with Aphasia Included in Stroke Trials? A Systematic Review and Narrative Synthesis by Eileen Vaughan and Molly X Manning in Clinical Rehabilitation

## Appendix 1 Inclusionary Criteria

HIERARCHY FOR RECORDING DECISIONS TO EXCLUDE CITATIONS: Language – Date - Population – Design – Article type – Intervention focus.

Population

– Adults aged 18 + years who have a diagnosis of stroke (+/− a diagnosis of aphasia secondary to stroke).

Design

– Quantitative trial study using randomisation with comparator conditions (e.g., RCT, stepped wedge RCT, cluster randomised trial).

Intervention focus/timing

– Cognitive, psychosocial, self-management, and creative arts interventions at any point post-onset.
– Interventions conducted in inpatient setting +/or ≤3 months post onset if there is a primary focus on cognition, health-related quality of life, psychosocial wellbeing, and/or self-management.
– Interventions targeting physical functioning only if dialogue-based +/or if cognition, health-related quality of life, psychosocial wellbeing, +/or self-management is a key focus.

Article type

– Studies from any geographical location, published in English in a peer-reviewed journal from 2016 to November 2022.

### Exclusion criteria

Population

– Children (<18 years).
– No diagnosis of stroke (including TIA).
– Mixed groups of trial participants, e.g., people with AND without a diagnosis of stroke.
– Caregivers of people with stroke.

Design

– Any observational quantitative study design (e.g., cohort, case control, pre-/post-test).
– Any study not using randomization to examine effectiveness of intervention versus comparator.
– Post-hoc or secondary analyses.
– Pilot RCT or proof of concept studies.
– Non-empirical including opinion pieces, commentaries, theoretical articles.
– Any review (systematic, narrative, qualitative).

Intervention focus/timing

– Interventions conducted in inpatient setting +/or ≤3 months post onset that is not dialogue-based. These include, for example, acupuncture.
– Non-dialogue-based interventions without primary focus on cognition, health-related quality of life, psychosocial wellbeing, +/or self-management. These include physical interventions like core stability, weight shift training, limb function, gait, or dysphagia.
– Aphasia/communication specific interventions.
– Medical, surgical, pharmacological interventions including Botox, needling, electromyography, brain stimulation, and secondary prevention.

Article type

– Treatment guidelines documents.
– Grey literature/not published in a peer-reviewed journal.

## Appendix 2 Electronic Database Search Strategy

**Table:**

Database	Search terms – JUNE 2021
*Embase*	(‘stroke’/exp OR (stroke* OR ‘cerebrovascular disease’):ti) AND (‘randomised controlled trial’/exp OR (‘randomi?ed controlled’ OR ‘cluster randomi?ation’ OR ‘stepped wedge’):ab,ti) AND (2016:py OR 2017:py OR 2018:py OR 2019:py OR 2020:py OR 2021:py OR 2022:py) AND ‘article'/it
*PubMed*	((“stroke”[mh] OR “cerebrovascular accident”[tiab] OR “cerebrovascular disease”[tiab])) AND (“randomized controlled”[tiab] OR “randomised controlled”[tiab] OR “cluster randomized”[tiab] OR “cluster randomised”[tiab] OR “cluster randomization”[tiab] OR “cluster randomisation”[tiab] OR “stepped wedge”[tiab])
*MEDLINE (Ovid)*	(((exp “cerebrovascular accident” OR exp “stroke” OR exp “cerebrovascular disease”).ab,ti.)) AND (exp “randomized controlled trial” OR (randomized controlled OR randomised controlled OR cluster randomized OR cluster randomised OR cluster randomisation OR cluster randomization OR stepped wedge))
Database	Updated Search terms NOVEMBER 2022
*Embase*	(‘stroke’/exp OR (stroke* OR ‘cerebrovascular disease’):ti) AND (‘randomised controlled trial’/exp OR (‘randomi?ed controlled’ OR ‘cluster randomi?ation’ OR ‘stepped wedge’):ab,ti) AND [20–06–2021]/sd
*PubMed*	((“stroke”[mh] OR “cerebrovascular accident”[tiab] OR “cerebrovascular disease”[tiab])) AND (“randomized controlled”[tiab] OR “randomised controlled”[tiab] OR “cluster randomized”[tiab] OR “cluster randomised”[tiab] OR “cluster randomization”[tiab] OR “cluster randomisation”[tiab] OR “stepped wedge”[tiab]) AND 2021/06/20 *Filters applied:* *from 2021/6/20 - 3000/12/12*
*MEDLINE (Ovid)*	(cerebrovascular accident or stroke or cerebrovascular disease).ti. AND (exp “randomized controlled trial” OR (randomized controlled OR randomised controlled OR cluster randomized OR cluster randomised OR cluster randomisation OR cluster randomization OR stepped wedge)) limit 15 to (english language and humans and yr="2021 -Current” and “all adult (19 plus years)” and journal article)
