# Supplementary material for: Are People with Aphasia Included in Stroke Trials? A Systematic Review and Narrative Synthesis
Source: Clin Rehabil. 2023 May 15;37(10):1375–85. doi: 10.1177/02692155231172009 (PMC10426244; doi:10.1177/02692155231172009)
Supplement: sj-pdf-2-cre-10.1177_02692155231172009 - Supplemental material for Are People with Aphasia Included in Stroke Trials? A Systematic Review and Narrative Synthesis [file sj-pdf-2-cre-10.1177_02692155231172009.pdf]

SUPPLEMENTARY FILE 2: STUDY CHARACTERISTICS AND METHODOLOGICAL APPRAISAL

	A	B	C	D	E	F	G	H	I	J	K	L	M	N	O	P	Q
1																	
2																	
3			**ELIGIBILITY**														
4	Reference	KEY INTERVENTION COMPONENTS<br>1 Physical (incl. significant cognitive or dual-tasking component +/or key cognitive or wellbeing outcome measure)<br>2 Cognition (e.g., attention, memory, reasoning, executive fn)<br>3 Self-management (incl. self-efficacy, adherence, health behaviour and sexual health)<br>4 Psychological wellbeing and health-related quality of life<br>5 MDT rehabilitation (with focus on any of 1-4)	Communication-Eligibility<br>1 = Functional communication description.<br>2 = Excludes severe aphasia (expressive and/or receptive and Wernicke's).<br>3 = Excudes moderate - severe aphasia.<br>4 = Excludes all aphasia and/or communication problem.<br>5 = Aphasia / Communication criteria not explicitly mentioned and/or are unclear (including those with MMSE, MOCA, NIHSS criteria).	**Communication-Eligibility detail**	**Communication Ax detail = 1**	**Communication Ax**	Did author discern any communication-relevant limitations due to eligibility criteria?<br>0 = No<br>1 = Yes	**Author-identified eligibility limitations**	Total participants	Reported number of participants with aphasia (despite any contradicting eligibility conditions)	Unclear if any PWA = 1 (e.g., when F3 is 5 BUT something in the text suggests that maybe PWA were included)	Inclusionary / retention strategies mentioned?<br>0 = No<br>1 = Yes	Number retained	percentage retained	Number retained not reported= 1	100% retained?<br>0 = No<br>1 = Yes	Does the article report reasons for attrition?<br>No = 0<br>Yes = 1
5																	
6	Adamit, T., Shames, J., & Rand, D. (2021, Jul 28). Effectiveness of the Functional and Cognitive	3	1	ability to understand and speak the language (Hebrew)			**0**		66			0	59	89			1
7	AIDAR, FJ et al 2016. A randomized trial investigating the influence of strength training on quality of life in ischemic stroke. Topics in Stroke	1	4	Excluded any aphasia.			0		29			0	24	83			**0**
8	An, H. S., & Kim, D. J. (2021). Effects of activities of daily living-based dual-task training on upper extremity function, cognitive function, and	2	4	Includes only those who had no issues in communication or problems with vision or hearing and could follow instructions.			0		30			0	30	100		1	**0**
9	APPALASAMY, J. R.et al 2020. An evaluation of the video narrative technique on the self-	3	5	Inclusion "satisfactory literacy...only those who were able to comprehend in English or			0		**216**			0	**167**	77			1
10	BALTADUONIENE, D et al 2019. Change of cognitive	2	4	Excluded "subjects unable to speak or have			0		126			0	121	96			**1**
11	BRAGSTAD, L. et al 2020. The effects of a dialogue-based intervention to promote psychosocial well-being after stroke: a randomized controlled	4	2	Excluded severe receptive / expressive aphasia (to ensure informed consent obtained - otherwise perceived as too time-consuming to include in the clinical setting).	1	Determined via The Ullevaal Aphasia Screening Test (UAS) with <50 cut-off.<br><br>Inferred from data in table 2, which	1	Identified difficulties enrolling patients with more severe stroke symptoms and aphasia and that this cohort may presumably be more vulnerable to psychosocial problems. The nurses and OT that enrolled participants reported	353	86		0	**282**	80			**1**

SUPPLEMENTARY FILE 2: STUDY CHARACTERISTICS AND METHODOLOGICAL APPRAISAL

	A	B	C	D	E	F	G	H	I	J	K	L	M	N	O	P	Q
12	Brouwer-Goossensen, D., Scheele, M., van Genugten, L., Lingsma, H. F., Dippel, D. W. J., Koudstaal, P. J., & den Hertog, H. M. (2022, Jan 11).	3	2	Excluded non-Dutch speaking; severe aphasia.			0		136	0		0	92	68		0	1
13	CHAN, W. N. & TSANG, W. W. N. 2018. The effect of Tai Chi training on the dual-tasking performance of stroke survivors: a randomized controlled trial. Clinical rehabilitation, 32, 1076-1085.	1	1	Inclusion: able to follow instructions in Cantonese.			0		26			0	17	65			1
14	CHEN, C.-H., et al2019. Mind-body interactive qigong improves physical and	1	1	Inclusion "able to communicate verbally".			0		72			0	68	94			1
15	Chen, Y., Wei, Y., Lang, H., Xiao, T., Hua, Y., Li, L., Wang, J., Guo, H., & Ni, C. (2021). Effects	3	4	Excludes aphasia / other language barriers.			1	"According to the inclusion/exclusion criteria, stroke patients with aphasia or cognitive impairment were excluded. It was both	96		1	1	89	93		0	1
16	CHOI, H.-S.et al 2019. Mirror Therapy Using Gesture Recognition for Upper Limb Function, Neck	1	1	Exclusion: "aphasia that makes intervention difficult".			0		36			0	36	100		1	0
17	DOUSSOULIN, Aet al 2017. Recovering functional independence after a stroke through Modified Constraint-Induced Therapy. NeuroRehabilitation, 40, 243-249.	1	5	Inclusion required score of 5 to 14 points on the NIH Stroke Scale; no aphasia-specific score required.	1	No aphasia score on NIHSS	0		36			0	36	100		1	0
18	FARIA, A. Let al2020. A comparison of two personalization and adaptive cognitive rehabilitation approaches: A randomized controlled trial with chronic stroke [illegible]	2	5	Exclusion: "Patients with a total score of more than two standard deviations below the mean score for age and education in the Montreal Cognitive Assessment [illegible]			0		32			0	19	59			1

SUPPLEMENTARY FILE 2: STUDY CHARACTERISTICS AND METHODOLOGICAL APPRAISAL

	A	B	C	D	E	F	G	H	I	J	K	L	M	N	O	P	Q
19	Feng, W., Yu, H., Wang, J., & Xia, J. (2021). Application effect of the hospital-community integrated service model in home rehabilitation of stroke in disabled elderly: a	3	5	Likely that PWA included because focus is on people with disabilities and impact of aphasia is acknowledged.			0		120		1	0		0	1		
20	FUJIOKA, T. et al 2018. The effects of music-supported therapy on motor, cognitive, and psychosocial functions in chronic	5	2	Exclusion included "no severe apraxia, aphasia...based on the self-report as well as observation during the screening visit".	1	self-report and observation during screening visit	0		29			1	28	97			0
21	Ge, C., Zhang, H., Zhu, G., Cao, A., & Zhang, J. (2021). Intervention study of Snyder's hope theory on the stigma of stroke in	4	5				0		94		1	0	94	100		1	0
22	Gjellesvik, T. I., Becker, F., Tjønna, A. E., Indredavik, B., Lundgaard, E., Solbakken, H., Brurok, B., Tørhaug, T., Lydersen, S., & Askim, T. (2021). Effects	1	5	"Another weakness is that our study lacks data on participants' educational levels, aphasia, visual deficits, and fine motor deficits, which are			1	"Another weakness is that our study lacks data on participants' educational levels, aphasia, visual deficits, and fine motor deficits, which are potential confounders when	70		1	0	56	80		0	1
23	Guillaumier, A., Spratt, N. J., Pollack, M., Baker, A., Magin, P., Turner, A., Oldmeadow, C., Collins, C., Callister, R., Levi, C., Searles, A.	4	5				1	"generalisability is limited by the sample comprising mostly 'well' stroke survivors. However, this trial provides a pathway for future testing and development of the program, with the	399			1	356	89			1
24	HAIRE, C. M., VUONG, V., TREMBLAY, L., PATTERSON, K. K., CHEN, J. L. & THAUT, M. H. 2021. Effects of therapeutic instrumental music performance and motor	2	1	Inclusion required "ability to understand and follow simple instructions".			0		30			0	30	100		1	0

SUPPLEMENTARY FILE 2: STUDY CHARACTERISTICS AND METHODOLOGICAL APPRAISAL

	A	B	C	D	E	F	G	H	I	J	K	L	M	N	O	P	Q
25	HILL, K., et al 2019. Prevention of mood disorder after stroke: A randomised controlled trial of problem solving therapy versus	4	1	Exclusion included: "unable to participate through impaired speech".			0		450			0	365	81			1
26	KANNAN, L.et al2019. Cognitive-motor exergaming for reducing fall risk in people with chronic stroke: A randomized controlled trial. NeuroRehabilitation, 44, 493-[illegible]	1	4	Inclusion: "without any presence of aphasia".			0		25			0	22	88			1
27	KONGKASUWAN et al 2016. Creative art therapy to enhance rehabilitation for stroke patients: a [illegible]	5	1	Inclusion: "could communicate verbally (answer question reasonably)".			0		118			0	113	96			1
28	KOOTKER, J. A.et al2017. Augmented Cognitive Behavioral Therapy for Poststroke Depressive Symptoms: A Randomized Controlled Trial. Arch Phys Med Rehabil, 98, [illegible]	4	5	Inclusion: Mini-Mental State Examination score >27 and "score positively on the communication-related items of" NIHSS.	1	NIHSS	0		61			0	46	75			0
29	Li, N., Wang, J., Zheng, M., & Ge, Q. (2021). Application Value of Rehabilitation Nursing in Patients with Stroke Based on the Theory of Interactive Standard: A Randomized Controlled Study [Article]. Evidence-Based Complementary and Alternative Medicine, 2021.	3	5				0		120	0	1	0	120	100		1	1

SUPPLEMENTARY FILE 2: STUDY CHARACTERISTICS AND METHODOLOGICAL APPRAISAL

	A	B	C	D	E	F	G	H	I	J	K	L	M	N	O	P	Q
30	Lin, S., Xiao, L. D., Chamberlain, D., Ullah, S., Wang, Y., Shen, Y., Chen, Z., & Wu, M. (2022, Apr). Nurse-led health coaching programme to improve hospital-to-home transitional care for stroke survivors: A randomised controlled trial. Patient Education and Counseling, 105(4), 917-925.	3	4	excludes " could speak Chinese without aphasia"			1	"Third, our study excluded participants with aphasia or cognitive impairment. Only first-time stroke survivors were included in the study. Therefore, the effectiveness of the health coaching intervention on stroke survivors with cognitive impair ment, aphasia or second-time stroke remains inconclusive."	140	0		0	134	96		0	1
31	LO, S. H et al (2018). Stroke self-management support improves survivors' self-efficacy and outcome [illegible]	3	5	Inclusion: score >18 in Mini Mental State Examination.			0		128			0	79	62			1
32	Mahmood, A., Nayak, P., English, C., Deshmukh, A., Shashikiran, U., Manikandan, N., & Solomon, J. [illegible]	3	1	able to communicate effectively			1	". The results of this study cannot be generalized to people with aphasia, cognitive impairments, or other neuropsy chiatric conditions. Hence, [illegible]	58		1	0	50	86			1
33	Matchar, D. B., Young, S. H. Y., Sim, R., Yu, C. J. Y., Yan, X., De Silva, D. A., & Chakraborty, B. (2022). Incentives for [illegible]	3	5	ex: "severe dysphasia without caregiver / proxy"			0		266		1	1	212	80			1
34	Mohammadi, E., Hassandoost, F., & Mozhdehipanah, H. (2022). Evaluation of the "partnership [illegible]	5	4	excludes "aphasia (perceptual or expressive"					80			1	67	84			1
35	NG, L. et al 2017. Effectiveness of a structured sexual rehabilitation programme	3	1	Inclusion: "ability to comprehend on Functional Independence Measure, FIM scale".	1	FIM scale comprehension	0		68			0	51	75			1
36	Niu, Y., Sheng, S., Chen, Y., Ding, J., Li, H., Shi, S., Wu, J., & Ye, D. (2022, Feb). The Efficacy of	4	4				1	"Besides, in patients with ... aphasia, the evaluation of depression symptoms is rather difficult and challenging, more	104			0	80	77			1

SUPPLEMENTARY FILE 2: STUDY CHARACTERISTICS AND METHODOLOGICAL APPRAISAL

	A	B	C	D	E	F	G	H	I	J	K	L	M	N	O	P	Q
37	Ozen, S., Senlikci, H. B., Guzel, S., & Yemisci, O. U. (2021). Computer Game	1	2	"sensory aphasia"					38		1	0	30	79			1
38	PANG, M. Y. C., et al2018. Dual-task exercise reduces cognitive-motor interference in walking and falls after stroke: A randomized controlled study. Stroke, 49, 2990-2998.	1	1	Inclusion: "ability to follow 3-step commands".			0		84			0	78	93			1
39	Pereira, F., Bermudez, I. B. S., Jorge, C., & Cameirao, M. S. (2021). The use of game modes to promote engagement and social involvement in multi-user serious games: a within-person randomized trial with stroke survivors [Randomized Controlled Trial Research Support, Non-U.S. Gov't]. Journal of Neuroengineering and Rehabilitation, 18(1), 62.	1	4	"any type of aphasia diagnosed" = excluded.	1	aphasia diagnosis			20			1	20	100		1	
40	RASMUSSEN, R. S et al 2016. Stroke rehabilitation at home before and after discharge reduced disability and improved quality of life: a randomised controlled trial. Clinical rehabilitation, 30, 225-236.	5	1	Exclusion: "Premorbidly unable to understand or speak the Danish language...severe memory impairments causing them to fail to understand and act upon instructions."			1	Primarily our study included inpatients with moderate stroke sequelae, and our results may not be valid for inpatients with severe or mild sequelae. Furthermore inpatients with profound aphasia were not included and present a limitation to our investigation.	71			0	61	86			1

SUPPLEMENTARY FILE 2: STUDY CHARACTERISTICS AND METHODOLOGICAL APPRAISAL

	A	B	C	D	E	F	G	H	I	J	K	L	M	N	O	P	Q
41	Rocha, L. S. O., Gama, G. C. B., Rocha, R. S. B., Rocha, L. B., Dias, C. P., Santos, L. L. S., Santos, M. C. S., Montebelo, M. I. L., & Teodori, R. M. (2021). Constraint Induced Movement Therapy Increases Functionality and Quality of Life after Stroke [Randomized Controlled Trial]. Journal of Stroke and Cerebrovascular Diseases, 30(6), 105774.	1	2	Not in eligibility BUT excluded n=5 with Wernicke's aphasia.					30		1	0	30	100		1	
42	Sakakibara, B. M., Lear, S. A., Barr, S. I., Goldsmith, C. H., Schneeberg, A., Silverberg, N. D., Yao, J., & Eng, J. J. (2022, Apr). Telehealth coaching to improve self-management for secondary prevention after stroke: A randomized controlled trial of Stroke Coach. International Journal of Stroke, 17(4), 455-464.	3	5					limitation that participants had mild stroke & high activity levels.	126		1	0	98	78			1
43	SIT, J. W. et al 2016. Do empowered stroke patients perform better at self-management and functional recovery after a stroke? A randomized controlled trial. Clinical interventions in aging, 11, 1441-1450.	3	4	"Stroke survivors with aphasia, cognitive impairment (mini-mental state examination score ,18) ...were excluded."			0		210			0	174	83			1

SUPPLEMENTARY FILE 2: STUDY CHARACTERISTICS AND METHODOLOGICAL APPRAISAL

	A	B	C	D	E	F	G	H	I	J	K	L	M	N	O	P	Q
44	SONG, C.-Set al. 2019. Cognitive strategy on upper extremity function for stroke: A randomized controlled trials. Restorative neurology and neuroscience, 37, 61-70.	1	1	Inclusion: "Sufficient cognitive ability to understand and follow simple verbal instructions".			0		50			0	49	98			0
45	Sylaja, P. N., Singh, G., Sivasambath, S., Arun, K., Jeemon, P., Antony, R., Kalani, R., Gopal, B. K., & Soman, B. (2021). Secondary prevention of stroke by a primary health care approach: An open-label cluster randomised trial [Randomized Controlled Trial]. Journal of Clinical Neuroscience, 84, 53-59.	3	5						238			0	234	98			1
46	TANG, A. et al 2016. High- and low-intensity exercise do not improve cognitive function after stroke: A randomized	1	5	"Data were missing for 3% of the cognitive data due to significant aphasia (n = 4) and difficulty understanding			0		50	5		0	47	94			0
47	Tao, J., Zhang, S., Kong, L., Zhu, Q., Yao, C., Guo, Q., Wu, J., Shan, C., & Fang, M. (2022). Effectiveness and functional magnetic	1	2	(can understand & follow protocol) AND excludes severe language impairment					84		1	0	39	46			1
48	Tarantino, V., Burgio, F., Toffano, R., Rigon, E., Meneghello, F., Weis, L., & Vallesi, A. (2021). Efficacy of a training on	2	2	severe comp deficits; dyslexia					37	16		0		0	1		

SUPPLEMENTARY FILE 2: STUDY CHARACTERISTICS AND METHODOLOGICAL APPRAISAL

	A	B	C	D	E	F	G	H	I	J	K	L	M	N	O	P	Q
49	Ünal Aslan, K. S., & Altın, S. (2022). Aromatherapy and foot massage on happiness, sleep quality, and fatigue levels in	4	4	"laceration or lesion on the foot, bone deformation in the foot, history of allergy, cognitive or mental or lingual problems,			0		115	0	0	0	91	79		0	0
50	Urcan, Z., & Kolcu, M. (2022, Feb). Effect of a Nurse-Led Education Program for Stroke Patients on Sleep Quality	3	4	"no communication problems"			0		120	0	0	1	92	77		1	0
51	VAN DE VEN, R. M et al 2017. The influence of computer-based cognitive flexibility training on subjective	2	1	Participants all had cognitive impairment. Inclusion: "still had cognitive complaints at study entry, and were able to work with a computer."			0		97			0	84	87			1
52	VISSER, M. M. et al 2016. Problem-Solving Therapy During Outpatient Stroke Rehabilitation Improves Coping and Health-Related Quality of Life: Randomized Controlled Trial. Stroke, 47, 135-42	4	3	Excluded those with "insufficient understanding of the Dutch language" and moderate or severe aphasia.			0		166			1	151	91			1
53	Vluggen, T., van Haastregt, J. C. M., Tan, F. E., Verbunt, J. A., van Heugten, C. M., & Schols, J. (2021). Effectiveness of an integrated multidisciplinary geriatric rehabilitation programme for older persons with stroke: a	5	5	They examined cognition by the MMSE but stated that they cannot rule out the fact that possible aphasic syndromes may have caused interference because they did not conduct a specific language assessment for stroke. Despite that, randomisation limited the			1	"there could have been interference by possible language disturbances caused by stroke. Although we examined cognition by the MMSE we cannot rule out the fact that possible aphasic syndromes may have caused interference because we did not conduct a specific language assessment for stroke. Despite that, randomisation	190	0	**1**	1	143	75	0	0	1

SUPPLEMENTARY FILE 2: STUDY CHARACTERISTICS AND METHODOLOGICAL APPRAISAL

	A	B	C	D	E	F	G	H	I	J	K	L	M	N	O	P	Q
54	WAN, L.-H.et al. 2016. Effectiveness of Goal-Setting Telephone Follow-Up on Health Behaviors of Patients with Ischemic Stroke: A Randomized Controlled Trial. Journal of stroke and cerebrovascular diseases : the official	3	2	Inclusion: "ability to communicate and provide informed consent". Exclusion: Diagnosis of Wernicke's and cognitive problems made by physician.	1	physician Dx	0		91			0	80	88			1
55	WICHOWICZ, H. M.,et al 2017. Application of Solution-Focused Brief Therapy (SFBT) in individuals after stroke. Brain Injury, 31, 1507-1512.	4	4	Exclusion: "The exclusion criteria were as follows: age over 65 years, aphasia, severe dysarthria".			0		62			0	24	39			1
56	Wilson, P. H., Rogers, J. M., Vogel, K., Steenbergen, B., McGuckian, T. B., & Duckworth, J. (2021, Nov 25). Home-based (virtual) rehabilitation improves motor and cognitive function for stroke patients: a randomized controlled trial of the Elements (EDNA-22) system. Journal of Neuroengineering and Rehabilitation, 18(1), 165. https://doi.org/10.1186/s12984-021-	1	1	(excludes those with the inability to follow and comprehend oral instructions			0		19	0	0	0	17	89	0	0	1

SUPPLEMENTARY FILE 2: STUDY CHARACTERISTICS AND METHODOLOGICAL APPRAISAL

	A	B	C	D	E	F	G	H	I	J	K	L	M	N	O	P	Q
57	Yan, L. L., Gong, E., Gu, W., Turner, E. L., Gallis, J. A., Zhou, Y., Li, Z., McCormack, K. E., Xu, L. Q., Bettger, J. P., Tang, S., Wang, Y., & Oldenburg, B. (2021). Effectiveness of a primary care-based integrated mobile health intervention for stroke management in rural China (SINEMA): A cluster-randomized controlled trial [Article]. PLoS Medicine, 18(4).	3	1	basic communication ability			0		1,299	0	0	0	1226	94		0	1
58	Yang, Y., Niu, G., Mi, Q., Hong, F., & Zhang, G. (2022). Analysis of Rehabilitation Effect of Neurology Nursing on Stroke Patients with Diabetes Mellitus and Its Influence on Quality of Life and Negative Emotion Score [Article]. Disease Markers, 2022.	3	5	n/a			0		110		1	0		0	1	0	0

SUPPLEMENTARY FILE 2: STUDY CHARACTERISTICS AND METHODOLOGICAL APPRAISAL

	A	B	C	D	E	F	G	H	I	J	K	L	M	N	O	P	Q
59	Yeh, T. T., Chang, K. C., Wu, C. Y., Chen, C. J., & Chuang, I. C. (2022, May). Clinical efficacy of aerobic exercise combined with computer-based cognitive training in stroke: a multicenter randomized controlled trial. Topics in Stroke Rehabilitation, 29(4), 255-264.	2	1	limited			0	n/a	56	0	0	0	56	100	0	1	
60	Yin, X. J., Wang, F., Lin, G. P., Gong, X. L., & Yao, M. Y. (2022, Aug). Effects of auricular acupressure on depression in stroke patients: A single-blind randomized controlled trial. Complementary Therapies in Clinical Practice, 48, 101596.	4	4	specifically stated excludes aphasia			0		60	0	0	0	56	93	0	0	1
61	Yu, J., Tang, Y., Han, J., Chen, J., Lin, W., & Cui, W. (2022). Reminiscence therapy is a feasible care program for improving cognitive function, anxiety, and depression in recurrent acute ischemic stroke patients: a randomized, controlled study [Article in Press]. Irish Journal of Medical Science.	2	5	Nil information re same			0	Nil	160	0	0	0	143	89	0	0	1

SUPPLEMENTARY FILE 2: STUDY CHARACTERISTICS AND METHODOLOGICAL APPRAISAL

	A	B	C	D	E	F	G	H	I	J	K	L	M	N	O	P	Q
62	Yuet Wong, F. K., Wang, S. L., Ng, S. S. M., Lee, P. H., Ching Wong, A. K., Li, H., Wang, W., Wu, L., Zhang, Y., & Shi, Y. (2022). Effects of a transitional home-based care program for stroke survivors in Harbin, China: a randomized controlled trial [Article]. Age and Ageing, 51(2).	5	5	limited			0		116	0	1	0	111	96	0	0	1

SUPPLEMENTARY FILE 2: STUDY CHARACTERISTICS AND METHODOLOGICAL APPRAISAL

**** CASP: 1 = yes; 2 = can't tell; 3 = no**

	A) Is the basic study design valid for a randomised controlled trial?			B) Was the study methodologically sound?					C) What are the results?			D) Will the results help locally?	
Reference	**1. Did the study address a clearly focused research question?** CONSIDER: Was the study designed to assess the outcomes of an intervention? Is the research question 'focused' in terms of: • Population studied • Intervention given • Comparator chosen • Outcomes measured?	**2. Was the assignment of participants to interventions randomised?** CONSIDER: • How was randomisation carried out? Was the method appropriate? • Was randomisation sufficient to eliminate systematic bias? • Was the allocation sequence concealed from investigators and participants?	**3. Were all participants who entered the study accounted for at its conclusion?** CONSIDER: • Were losses to follow-up and exclusions after randomisation accounted for? • Were participants analysed in the study groups to which they were randomised (intention-to-treat analysis)? • Was the study stopped early? If so, what was the reason?	**4A** • Were the participants 'blind' to intervention they were given?	**4B** • Were the investigators 'blind' to the intervention they were giving to participants?	**4C** • Were the people assessing/analysing outcome/s 'blinded'?	**5. Were the study groups similar at the start of the randomised controlled trial?** CONSIDER: • Were the baseline characteristics of each study group (e.g. age, sex, socio-economic group) clearly set out? • Were there any differences between the study groups that could affect the outcome/s?	**6. Apart from the experimental intervention, did each study group receive the same level of care (that is, were they treated equally)?** CONSIDER: • Was there a clearly defined study protocol? • If any additional interventions were given (e.g. tests or treatments), were they similar between the study groups? • Were the follow-up intervals the same for each study group?	**7. Were the effects of intervention reported comprehensively?** CONSIDER: What outcomes were measured, and were they clearly specified? How were the results expressed? For binary outcomes, were relative and absolute effects reported? Were the results reported for each outcome in each study group at each follow-up interval? Was there any missing or incomplete data? Was there differential drop-out between the study groups that could affect the results? Were potential sources of bias identified? Which statistical tests were	**8. Was the precision of the estimate of the intervention or treatment effect reported?** CONSIDER: • Were confidence intervals (CIs) reported?	**9. Do the benefits of the experimental intervention outweigh the harms and costs?** CONSIDER: • What was the size of the intervention or treatment effect? • Were harms or unintended effects reported for each study group? • Was a cost-effectiveness analysis undertaken? (Cost-effectiveness analysis allows a comparison to be made between different interventions used in the care of the same condition or problem.)	**10. Can the results be applied to your local population/in your context?** CONSIDER: • Are the study participants similar to the people in your care? • Would any differences between your population and the study participants alter the outcomes reported in the study? • Are the outcomes important to your population? • Are there any outcomes you would have wanted information on that have not been studied or reported? • Are there any limitations of the study	**11. Would the experimental intervention provide greater value to the people in your care than any of the existing interventions?** CONSIDER: • What resources are needed to introduce this intervention taking into account time, finances, and skills development or training needs? • Are you able to disinvest resources in one or more existing interventions in order to be able to re-invest in the new intervention?
Adamit, T., Shames, J., & Rand, D. (2021, Jul 28). Effectiveness of the Functional and Cognitive	1	1	1	3	3	1	1	1	1	1	2	3	2
AIDAR, FJ et al 2016. A randomized trial investigating the influence of strength training on quality of life in ischemic stroke. Topics in Stroke	1	2	1	3	3	1	2	1	3	3	2	3	3
An, H. S., & Kim, D. J. (2021). Effects of activities of daily living-based dual-task training on upper extremity function, cognitive	1	2	1	3	3	3	1	1	1	3	2	3	2
APPALASAMY, J. R.et al 2020. An evaluation of the video narrative technique on	1	1	1	3	3	1	3	1	1	1	2	3	2
BALTADUONIENE, D et al 2019. Change	1	1	1	1	3	3	1	1	1	1	2	3	3
BRAGSTAD, L. et al 2020. The effects of a dialogue-based intervention to promote psychosocial well-being after stroke: a randomized controlled	1	1	1	3	3	1	3	1	1	1	3	3	3
Brouwer-Goossensen, D., Scheele, M., van Genugten, L., Lingsma, H. F., Dippel, D. W. J., Koudstaal, P. J., & den Hertog, H. M. (2022, Jan	1	1	1	2	2	1	1	1	1	1	2	2	3

SUPPLEMENTARY FILE 2: STUDY CHARACTERISTICS AND METHODOLOGICAL APPRAISAL

CHAN, W. N. & TSANG, W. W. N. 2018. The effect of Tai Chi training on the dual-tasking performance of stroke survivors: a randomized controlled trial. Clinical rehabilitation, 32, 1076-[illegible]	1	2	1	3	3	1	1	1	1	2	2	3	2
CHEN, C.-H., et al2019. Mind-body interactive qigong improves physical and	1	1	1	3	3	3	2	1	2	3	2	2	2
Chen, Y., Wei, Y., Lang, H., Xiao, T., Hua, Y., Li, L., Wang, J., Guo, H., & Ni, C. (2021).	1	1	1	1	3	1	1	1	1	3	2	2	2
CHOI, H.-S.et al 2019. Mirror Therapy Using Gesture Recognition for Upper Limb Function.	1	1	1	3	3	1	1	1	1	3	2	3	2
DOUSSOULIN, Aet al 2017. Recovering functional independence after a stroke through Modified Constraint-Induced Therapy. NeuroRehabilitation, 40, 243-249.	2	2	1	2	3	1	3	1	3	3	2	3	2
FARIA, A. Let al2020. A comparison of two personalization and adaptive cognitive rehabilitation approaches: A randomized controlled trial with chronic stroke	2	1	1	3	3	3	1	1	1	1	2	2	2
Feng, W., Yu, H., Wang, J., & Xia, J. (2021). Application effect of the hospital-community integrated service model in home rehabilitation of stroke in disabled elderly: a	3	2		2	2	2	2	2	3	3	2	3	3
FUJIOKA, T. et al 2018. The effects of music-supported therapy on motor, cognitive, and psychosocial functions in chronic	1	1	1	3	3	1	2	1	1	3	2	3	3
Ge, C., Zhang, H., Zhu, G., Cao, A., & Zhang, J. (2021). Intervention study of Snyder's hope theory on the stigma of stroke in	3	2	1	2	2	2	3	2	3	3	2	3	3

SUPPLEMENTARY FILE 2: STUDY CHARACTERISTICS AND METHODOLOGICAL APPRAISAL

Gjellesvik, T. I., Becker, F., Tjønna, A. E., Indredavik, B., Lundgaard, E., Solbakken, H., Brurok, B., Tørhaug, T., Lydersen, S., & Askim, T. (2021)	1	1	1	2	3	1	1	1	1	1	2	3	2
Guillaumier, A., Spratt, N. J., Pollack, M., Baker, A., Magin, P., Turner, A., Oldmeadow, C., Collins, C., Callister, R., Levi, C., Searles, A [illegible]	1	1	1	2	3	1	1	1	1	1	1	2	2
HAIRE, C. M., VUONG, V., TREMBLAY, L., PATTERSON, K. K., CHEN, J. L. & THAUT, M. H. 2021. Effects of therapeutic instrumental music performance	2	1	1	3	3	1	2	1	1	3	2	2	2
HILL, K., et al 2019. Prevention of mood disorder after stroke: A randomised controlled trial of problem solving	1	1	1	3	3	1	2	1	1	1	2	3	2
KANNAN, L.et al2019. Cognitive-motor exergaming for reducing fall risk in people with chronic stroke: A randomized controlled trial. NeuroRehabilitation, 44, [illegible]	1	3	1	3	3	2	1	1	1	3	2	3	3
KONGKASUWAN et al 2016. Creative art therapy to enhance rehabilitation for stroke patients: a [illegible]	1	1	1	3	3	1	1	1	1	3	2	3	2
KOOTKER, J. A.et al2017. Augmented Cognitive Behavioral Therapy for Poststroke Depressive Symptoms: A Randomized Controlled Trial. Arch Phys Med Rehabil, 98, [illegible]	1	1	1	3	3	1	2	1	1	1	2	3	2
Li, N., Wang, J., Zheng, M., & Ge, Q. (2021). Application Value of Rehabilitation Nursing in Patients with Stroke Based on the Theory of Interactive Standard: A Randomized Controlled Study [Article]. Evidence-Based Complementary and Alternative Medicine, 2021.	3	2		3	3	2	2	1	3	3	3	3	3

SUPPLEMENTARY FILE 2: STUDY CHARACTERISTICS AND METHODOLOGICAL APPRAISAL

Lin, S., Xiao, L. D., Chamberlain, D., Ullah, S., Wang, Y., Shen, Y., Chen, Z., & Wu, M. (2022, Apr). Nurse-led health coaching programme to improve hospital-to-home transitional care for stroke survivors: A randomised controlled trial. Patient Education and Counseling, 105(4), 917-925.	1	1	1	3	3	2	2	1	1	2	1	2	2
LO, S. H et al (2018). Stroke self-management support improves survivors' self-efficacy and outcome [illegible]	1	1	1	3	3	1	3	1	3	1	2	2	2
Mahmood, A., Nayak, P., English, C., Deshmukh, A., Shashikiran, U., Manikandan, N., & Solomon, J. [illegible]	1	1	1	3	3	1	2	1	1	1	1	2	2
Matchar, D. B., Young, S. H. Y., Sim, R., Yu, C. J. Y., Yan, X., De Silva, D. A., & Chakraborty, B. (2022). Incentives for [illegible]	1	1	1	3	3	2	2	1	2	2	2	3	3
Mohammadi, E., Hassandoost, F., & Mozhdehipanah, H. (2022). Evaluation of the "partnership [illegible]	1	1	1	1	3	3	1	1	1	1	2	2	2
NG, L. et al 2017. Effectiveness of a structured sexual rehabilitation	1	1	1	3	3	1	1	1	1	1	2	3	2
Niu, Y., Sheng, S., Chen, Y., Ding, J., Li, H., Shi, S., Wu, J., & Ye, D. (2022, Feb). The Efficacy	1	1	1	2	2	2	1	1	1	3	2	2	2
Ozen, S., Senlikci, H. B., Guzel, S., & Yemisci, O. U. (2021). Computer Game	1	3	1	2	3	1	1	1	1	3	2	2	2
PANG, M. Y. C., et al2018. Dual-task exercise reduces cognitive-motor interference in walking and falls after stroke: A randomized controlled study. Stroke, 49, 2990-2998.	1	1	1	3	3	1	1	1	1	1	2	2	2

SUPPLEMENTARY FILE 2: STUDY CHARACTERISTICS AND METHODOLOGICAL APPRAISAL

Pereira, F., Bermudez, I. B. S., Jorge, C., & Cameirao, M. S. (2021). The use of game modes to promote engagement and social involvement in multi-user serious games: a within-person randomized trial with stroke survivors [Randomized Controlled Trial Research Support, Non-U.S. Gov't]. Journal of Neuroengineering and Rehabilitation, 18(1), 62.	1	2	2	2	3	2	2	1	1	3	2	3	3
RASMUSSEN, R. S et al 2016. Stroke rehabilitation at home before and after discharge reduced disability and improved quality of life: a randomised controlled trial. Clinical rehabilitation, 30, 225-236.	1	1	1	3	3	3	2	1	1	3	2	3	2
Rocha, L. S. O., Gama, G. C. B., Rocha, R. S. B., Rocha, L. B., Dias, C. P., Santos, L. L. S., Santos, M. C. S., Montebelo, M. I. L., & Teodori, R. M. (2021). Constraint Induced Movement Therapy Increases Functionality and Quality of Life after Stroke [Randomized Controlled Trial]. Journal of Stroke and Cerebrovascular Diseases, 30(6), 105774.	1	1	1	1	1	1	1	1	1	2	2	3	2
Sakakibara, B. M., Lear, S. A., Barr, S. I., Goldsmith, C. H., Schneeberg, A., Silverberg, N. D., Yao, J., & Eng, J. J. (2022, Apr). Telehealth coaching to improve self-management for secondary prevention after stroke: A randomized controlled trial of Stroke Coach. International Journal of Stroke, 17(4), 455-464.	1	1	1	3	3	1	1	1	1	1	3	2	3

SIT, J. W. et al 2016. Do empowered stroke patients perform better at self-management and functional recovery after a stroke? A randomized controlled trial. Clinical interventions in aging, 11, 1441-1450.	1	1	1	3	3	1	2	1	1	1	2	3	2
SONG, C.-Set al. 2019. Cognitive strategy on upper extremity function for stroke: A randomized controlled trials. Restorative neurology and neuroscience, 37, 61-70.	1	1	1	1	3	1	1	1	1	1	2	2	2
Sylaja, P. N., Singh, G., Sivasambath, S., Arun, K., Jeemon, P., Antony, R., Kalani, R., Gopal, B. K., & Soman, B. (2021). Secondary prevention of stroke by a primary health care approach: An open-label cluster randomised trial [Randomized Controlled Trial]. Journal of Clinical Neuroscience, 84, 53-59.	1	1	1	2	2	2	1	1	1	1	2	2	2
TANG, A. et al 2016. High- and low-intensity exercise do not improve cognitive function after stroke: A randomized	1	1	1	3	3	1	1	1	1+AA46	1	3	3	3
Tao, J., Zhang, S., Kong, L., Zhu, Q., Yao, C., Guo, Q., Wu, J., Shan, C., & Fang, M. (2022). Effectiveness and functional magnetic	1	1	1	3	3	1	1	1	1	1	2	2	2
Tarantino, V., Burgio, F., Toffano, R., Rigon, E., Meneghello, F., Weis, L., & Vallesi, A. (2021). Efficacy of a training on	1	1	2	1	3	3	1	1	1	3	2	2	2
Ünal Aslan, K. S., & Altın, S. (2022). Aromatherapy and foot massage on happiness, sleep quality, and fatigue levels in	1	1	1	1	3	1	1	1	3	1	1	1	3

SUPPLEMENTARY FILE 2: STUDY CHARACTERISTICS AND METHODOLOGICAL APPRAISAL

Urcan, Z., & Kolcu, M. (2022, Feb). Effect of a Nurse-Led Education Program for Stroke Patients on Sleep Quality	1	1	1	3	3	3	1	3	1	3	2	1	2
VAN DE VEN, R. M et al 2017. The influence of computer-based cognitive flexibility training on subjective	1	1	1	1	3	1	1	1	1	1	3	3	3
VISSER, M. M. et al 2016. Problem-Solving Therapy During Outpatient Stroke Rehabilitation Improves Coping and Health-Related Quality of Life: Randomized Controlled Trial. Stroke, 47, 135-42	1	1	1	3	3	1	1	1	1	1	1	3	3
Vluggen, T., van Haastregt, J. C. M., Tan, F. E., Verbunt, J. A., van Heugten, C. M., & Schols, J. (2021). Effectiveness of an integrated multidisciplinary geriatric rehabilitation programme for older persons with stroke: a	1	1	1	3	3	1	1	1	1	3	2	1	2
WAN, L.-H.et al. 2016. Effectiveness of Goal-Setting Telephone Follow-Up on Health Behaviors of Patients with Ischemic Stroke: A Randomized Controlled Trial. Journal of stroke and cerebrovascular diseases : the official	1	1	1	3	3	1	1	1	1	1	2	3	2
WICHOWICZ, H. M.,et al 2017. Application of Solution-Focused Brief Therapy (SFBT) in individuals after stroke. Brain Injury, 31, 1507-1512.	1	2	1	3	3	1	1	1	1	3	2	3	2

Wilson, P. H., Rogers, J. M., Vogel, K., Steenbergen, B., McGuckian, T. B., & Duckworth, J. (2021, Nov 25). Home-based (virtual) rehabilitation improves motor and cognitive function for stroke patients: a randomized controlled trial of the Elements (EDNA-22) system. Journal of Neuroengineering and Rehabilitation, 18(1), 165. https://doi.org/10.1186/s12984-021-	1	1	1	1	3	2	1	1	1	3	2	2	2
Yan, L. L., Gong, E., Gu, W., Turner, E. L., Gallis, J. A., Zhou, Y., Li, Z., McCormack, K. E., Xu, L. Q., Bettger, J. P., Tang, S., Wang, Y., & Oldenburg, B. (2021). Effectiveness of a primary care-based integrated mobile health intervention for stroke management in rural China (SINEMA): A cluster-randomized controlled trial [Article]. PLoS Medicine, 18(4).	1	1	3	1	1	1	1	1	1	1	1	1	2
Yang, Y., Niu, G., Mi, Q., Hong, F., & Zhang, G. (2022). Analysis of Rehabilitation Effect of Neurology Nursing on Stroke Patients with Diabetes Mellitus and Its Influence on Quality of Life and Negative Emotion Score [Article]. Disease Markers, 2022.	1	1	2	3	3	1	1	3	3	1	1	3	3

SUPPLEMENTARY FILE 2: STUDY CHARACTERISTICS AND METHODOLOGICAL APPRAISAL

Yeh, T. T., Chang, K. C., Wu, C. Y., Chen, C. J., & Chuang, I. C. (2022, May). Clinical efficacy of aerobic exercise combined with computer-based cognitive training in stroke: a multicenter randomized controlled trial. Topics in Stroke Rehabilitation, 29(4), 255-264.	1	1	1	3	1	1	1	1	1	3	2	2	2
Yin, X. J., Wang, F., Lin, G. P., Gong, X. L., & Yao, M. Y. (2022, Aug). Effects of auricular acupressure on depression in stroke patients: A single-blind randomized controlled trial. Complementary Therapies in Clinical Practice, 48, 101596.	1	1	1	1	3	3	1	1	1	3	2	3	3
Yu, J., Tang, Y., Han, J., Chen, J., Lin, W., & Cui, W. (2022). Reminiscence therapy is a feasible care program for improving cognitive function, anxiety, and depression in recurrent acute ischemic stroke patients: a randomized, controlled study [Article in Press]. Irish Journal of Medical Science.	1	1	1	3	3	3	1	1	1	3	2	2	2
Yuet Wong, F. K., Wang, S. L., Ng, S. S. M., Lee, P. H., Ching Wong, A. K., Li, H., Wang, W., Wu, L., Zhang, Y., & Shi, Y. (2022). Effects of a transitional home-based care program for stroke survivors in Harbin, China: a randomized controlled trial [Article]. Age and Ageing, 51(2).	1	1	1	1	1	1	1	1	1	3	2	3	3
